# Regional intra-arterial vs. systemic chemotherapy for the treatment of advanced pancreatic cancer: a systematic review and meta-analysis

**DOI:** 10.3389/fonc.2024.1197424

**Published:** 2024-04-08

**Authors:** Yanjie Cao, Dedong Yu, Yun Wu, Wei Zhu

**Affiliations:** Department of Oncology, Baotou Central Hospital, Baotou, China

**Keywords:** systemic chemotherapy, pancreatic cancer, partial remission, meta-analysis, regional intra-arterial chemotherapy

## Abstract

**Introduction:**

Pancreatic cancer is a highly aggressive malignancy with limited response to chemotherapy. This research aims to compare the effectiveness and safety of regional intra-arterial chemotherapy (RIAC) with conventional systemic chemotherapy in treating advanced stages of pancreatic cancer.

**Methods:**

A comprehensive literature review was conducted using databases such as PubMed, Embase, Web of Science, and the Cochrane Library. Studies assessing the comparative outcomes of RIAC and systemic chemotherapy were included. Data extraction and quality evaluation were performed independently by two researchers. Statistical analysis was conducted using STATA16 software, calculating odds ratios (OR), risk differences (RD), and 95% confidence intervals (CI).

**Results:**

Eleven studies, comprising a total of 627 patients, were included in the meta-analysis. The findings showed that patients undergoing RIAC had significantly higher rates of partial remission (PR) compared to those receiving systemic chemotherapy (OR = 2.23, 95% CI: 1.57, 3.15, I2= 0%). Additionally, the rate of complications was lower in the RIAC group (OR = 0.45, 95% CI: 0.33, 0.63, I2= 0%). Moreover, patients treated with RIAC had notably longer median survival times.

**Discussion:**

The results of this research indicate that RIAC is associated with a higher rate of partial remission, improved clinical benefits, and fewer complications compared to systemic chemotherapy in the management of advanced pancreatic cancer. These findings suggest that RIAC may be a more effective and safer treatment option for patients with advanced stages of pancreatic cancer.

**Systematic review registration:**

https://www.crd.york.ac.uk/prospero/, identifier CRD42023404637.

## Introduction

Pancreatic cancer is a malignant digestive tract tumor with an extremely high degree of malignancy and rapid progression. Pancreatic cancer patients usually have poor prognosis (1), and the 5-year overall survival rate is approximately 10% in the USA (2). Also, in recent years, the incidence rate of pancreatic cancer has been on the rise (3). Radical surgery remains the most effective approach, and the 5-year survival rate after surgery is about 20% (4, 5). However, considering that 85-90% of patients present with advanced tumors at the time of diagnosis, other treatment methods must be selected. Adjuvant chemotherapy has been recommended for patients with advanced pancreatic cancer. For example, gemcitabine (GEM), which is given systemically, is effective as postoperative adjuvant chemotherapy in patients with stage IV pancreatic cancer, with a response rate of only 5-15% (6). GEM does not significantly improve survival when combined with other anti-cancer drugs (7, 8). Still, some studies have shown that certain patients do not respond well to conventional systemic intravenous chemotherapy (9). On the other hand, conventional radiotherapy and chemotherapy for pancreatic cancer have limited effects, with an average survival time of 6 months (10).

In recent years, RAIC has been clinically used as a new chemotherapy regimen for advanced pancreatic cancer (9, 11). Pancreatic cancer is a retroperitoneal tumor lacking blood supply. RAIC delivers antineoplastic drugs to the tumor site through the ductus arteriosus, producing locally high drug concentrations while maintaining low systemic drug levels. Compared with conventional systemic intravenous chemotherapy, RAIC can improve the effect of the drug and reduce the appearance of adverse events in patients with colorectal cancer and liver metastases (12). Fang et al. (13) reported a clinical benefit of RIAC of 78.06% for patients with advanced pancreatic cancer, compared to 29.37% for those who received systemic chemotherapy. Also, the one-year survival rate for RIAC (28.6%) was higher than for systemic chemotherapy (0%) (13). Thus, it is believed that RIAC can improve the clinical benefit and survival rates in patients with advanced pancreatic cancer (14–16).

To the best of our knowledge, an increasing number of investigations have explored the efficacy of RIAC in advanced pancreatic cancer over the last few years (9, 17, 18). Nevertheless, the value of RIAC in treating advanced pancreatic has not been conclusively demonstrated. In addition, most of these studies remain in the phase II clinical trial stage, lacking comprehensive subgroup analysis of clinical research subjects. Considering the small number of patients included in the published studies and that most of the studies were retrospective, a systematic review and meta-analysis are necessary to provide a more reliable conclusion to guide clinical practice.

Herein, we used systematic review and meta-analysis to clarify the value of RIAC in treating advanced pancreatic cancer by comparing the safety and efficacy of RIAC with systemic chemotherapy.

## Materials and methods

This systematic review was conducted in accordance with the standards of the Preferred Reporting Items for Systematic Reviews and Meta-Analyses (PRISMA) (19). No ethical approval or informed consent was required for this article because all data were retrieved from published literature.

### Search strategy

Four electronic databases, i.e., PubMed, Embase, Web of Science, and Cochrane Library, were searched on May 30, 2023, and no time limitation was applied. Two investigators performed searching, identification of eligibility, data extraction, and quality assessment; disagreements were resolved through discussion. Vocabulary and syntax were specifically adapted according to the database. The specific search terms were: ((pancreatic or pancreas), (cancer or neoplasms or carcinoma or malignant tumor)), ((arteries or arterial) and (infusion or perfusion or chemotherapy)). Only studies published in the English language were included. Reference lists of relevant articles were also manually screened for additional possible records.

### Inclusion criteria

Studies were selected based on the following inclusion criteria (1): study design: randomized controlled trials (RCTs) (2); population: adult patients who were histologically and/or clinically diagnosed with pancreatic cancer (3); intervention: RIAC (given via cancer feeding artery, hepatic artery, celiac artery, gastroduodenal artery, superior mesenteric artery, common hepatic artery, splenic artery, or other regional arteries, with or without regional embolization), or systemic intravenous chemotherapy (given via central or peripheral veins) (4); outcomes: provided 1 of the following outcome of interest: complete remission (CR), partial remission (PR), or complications (5); sufficient data could be extracted. If more than one study provided overlapping data, only the latest study or a study with the most comprehensive data was included. Case reports, commentaries, expert opinions, and narrative reviews were excluded.

### Data extraction

Requisite data extracted and recorded to standardized Excel files included the first author’s surname, publication year, study inclusion interval, country, study design, demographic information of participants, number of patients in the RIAC and systemic treatment groups, gender, and route of drug administration. The primary endpoints were: complete response (CR), partial response (PR), and complication rate where CR indicated a disappearance of all target lesions [any pathological lymph nodes (whether target or non-target) must have reduction in short axis to <10 mm] and PR indicated at least a 30% decrease in the sum of diameters of target lesions, taking as reference the baseline sum diameters, with no evidence of new or progressive lesions. Side effects of interest mainly involved hematology (leukopenia, thrombocytopenia, or anemia) and gastrointestinal system complications (nausea, vomiting, or duodenal ulcers); other complications were embolism, thrombophlebitis, and catheter displacement.

### Quality assessment

The quality of the included non-RCTs was assessed by using the risk of bias in non-randomized studies of interventions (ROBINS-I). The RCTs were evaluated using the Cochrane risk bias tool 2.0.

### Statistical analyses

The heterogeneity between studies was assessed using Chi-square statistics and qualified by the size of I^2^. Heterogeneity among included studies was assessed using the I^2^ statistic. An I^2^ value of 0% implied no observed heterogeneity, and values of > 50% indicated substantial heterogeneity. All meta-analyses used a fixed-effects model: I^2^ < 25% for all accessed outcomes. The analyzed parameters included the number of patients, major endpoints (CR, PR, and complication rate), and side effects. The value of a two-sided *P*< 0.05 was considered statistically significant. Stata version 16 (StataCorp, College Station, TX, USA) was used to analyze data from RCTs meeting inclusion criteria. The potential publication bias was examined using Egger’s tests and funnel plot. Finally, sensitivity analyses were performed to identify individual study effects on pooled results and test the reliability of the results.

## Results

### Search results and study selection

**Figure 1:**
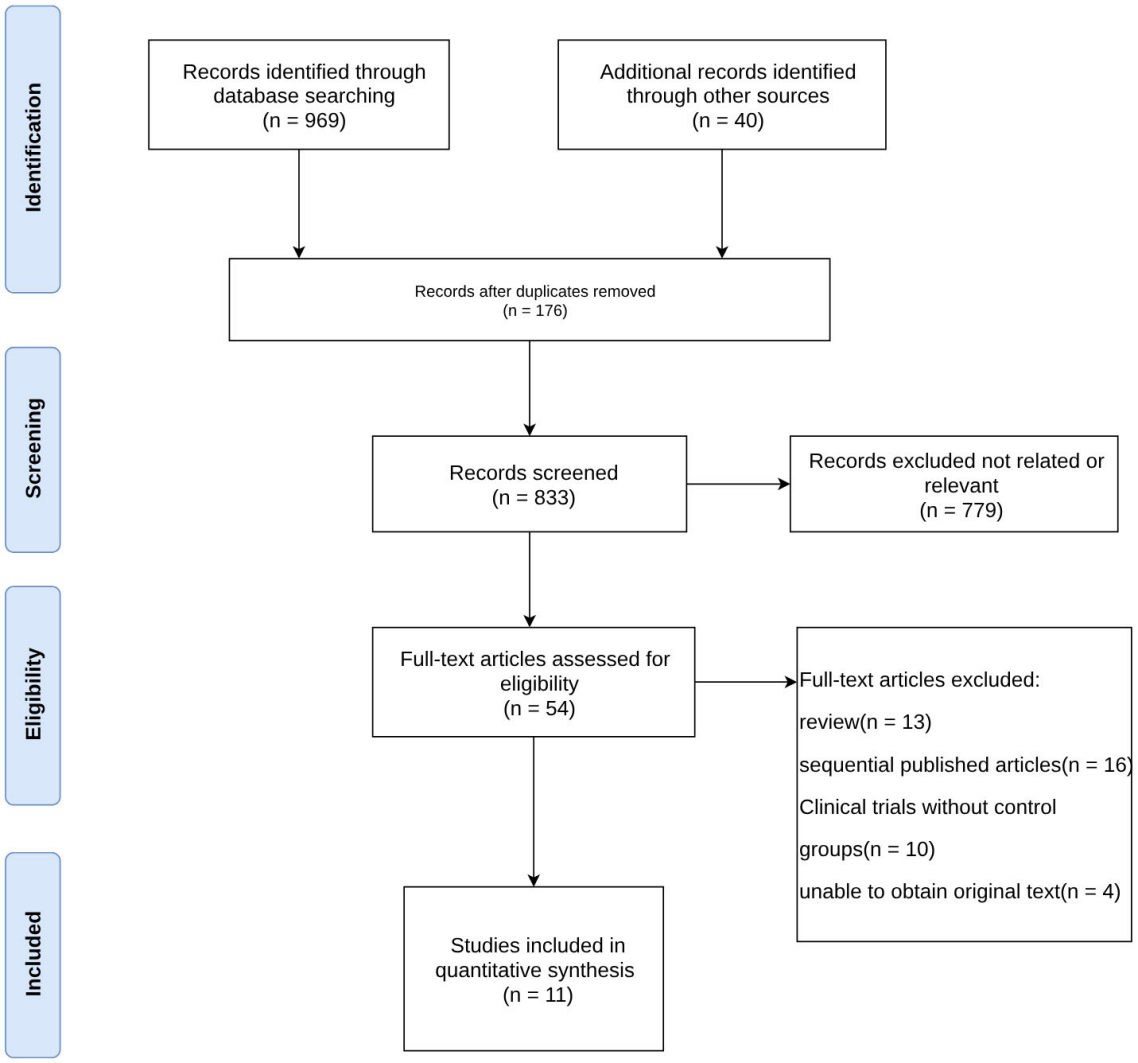
Selection process of included studies.

A total of 969 relevant papers were obtained from the preliminary search. There were 833 potentially relevant studies after excluding duplicates. After performing an initial screening of the title and abstract, 54 articles with strong correlations were obtained. Eleven articles (20–30) were finally included in the meta-analysis after assessing the full-text content and analyzing the data integrity according to the exclusion criteria. **Figure 1** shows the selection process of the included studies.

### Study characteristics

Eleven studies involved 627 patients, 322 of whom received regional intra-arterial chemotherapy, and 305 received systemic chemotherapy. The mean ages of included patients ranged from 55.0 to 62.4 years, and the proportion of males ranged from 52.9% to 57.1%. The chemotherapy regimen included FAM [adriamycin 40 mg/m^2^, mitomycin (MMC) 6 mg/m^2^, and 5-fluorouracil (5-FU) 375 mg/m^2^], GEM (1000 mg/m^2^), MF [MMC 2 mg, 5-FU 750 mg], MmMC [mitomycin C at a total dose of 18 mg/m^2^, mitoxantrone 6 mg/m^2^, and cisplatin 30 mg/m^2^]. GP (gemcitabine 1000 mg/m^2^, cisplatin 50 mg/m^2^), and GF (GEM+5-FU)[GEM 1000 mg/m^2^; 5-Fu, 600 mg/m^2^]. The drug delivery routes included celiac artery splenicartery, tumor-feeding arteries, splenic artery, gastroduodenal artery, common hepatic artery, and superior mesenteric artery (**Table 1**). Of note, the study by Wang et al. did not report the information on survival time (so the survival time in **Table 1** for this study is empty); yet, this study was included ins the meta-analysis because it met the inclusion criteria and reported the necessary data for meta-analysis.

**Table 1:** Characteristics of studies included in the meta-analysis.

Author	Country	Sample size	Diagnosis	Mean age	Drugs	Drug delivery routes	Side effects	Survival time (median or mean, interval)	Survival time (median or mean, interval)	Follow-up time (months)
		RIAC/SC						RIAC	SC	
Ji et al., 2003 (28)	China	18\11	pathological/CT/CA199/MRI	62.4	MF	splenic artery, gastroduodenal artery, common hepatic artery	cytopenia (mild), nausea and anorexia (mild), abscess around the pump (mild)	12.5	4.8	>1
Shamseddine et al., 2005 (29)	America	7\4	Biopsy proven	NR	GEM	tumor-feeding arteries	pain and fever (mild)	5	5.6	>28
Han et al., 2006 (27)	China	70\70	Biopsy proven	60.2	FAM	celiac artery splenic artery	nausea and vomiting (48.6% in RIAC/41.3% in SC); three severe myelosuppression and one death in SC	13.5 (3–34 months)	6.2 (1–13 months)	1-34
Hong et al., 2007 (30)	China	25\26	pathological/CT/MRI	NR	GF	tumor-feeding arteries	leukopenia, thrombocytopenia, anemia, nausea/vomiting, arrhythmia, aminotransferase (mild to moderate)	10	7.3	1-12
Liu et al., 2007 (24)	China	32\33	pathological/CT/MRI	55	GF	tumor-feeding arteries	leukopenia, thrombocytopenia, renal hypoplasia (mild)	11	8	0-140
Hong et al., 2008	China	25\26	pathological/CT/MRI	NR	GF	superior mesenteric artery	leukopenia, thrombocytopenia, anemia, nausea and vomiting(mild to moderate), arrhythmia, aminotransferase(mild)	12	6	0-30
Liu et al., 2008 (23)	China	26\27	pathological/CT/MRI	61	GP	superior mesenteric artery	leukopenia (severe, 2 in RIAC, 3 in SC), thrombocytopenia, nausea and vomiting, hepatic and renal hypoplasia (mild)	21(10.5–28 months)	14(5–20.5 months)	>28
Jia et al., 2009 (22)	China	21\22	pathological/CT/MRI	60	GF	superior mesenteric artery	leukopenia, thrombocytopenia, nausea and vomiting, constipation, hepatic and renal hypoplasia (mild to moderate), oral mucosal reactions (mild)	13.5	6.2	NR
Qi et al., 2011 (25)	China	36\32	pathological/CT/CA199/MRI	62	GP	splenic artery, gastroduodenal artery, common hepatic artery	leukopenia, thrombocytopenia, nausea and vomiting, hepatic and renal hypoplasia (mild)	12	8	24
Wang et al., 2012 (26)	China	38\30	pathological/CT/CA199/MRI	58.7	GF	gastroduodenal artery, common hepatic artery	nausea and vomiting(mild to moderate, two severe in SC), leukopenia, thrombocytopenia(four severe in RIAC, one in SC)	NR	–	NR
Hong et al., 2012 (20)	China	24\24	pathological	59.5	GF	abdominal cavity artery and upper mesentery artery	leukopenia, thrombocytopenia, nausea and vomiting, arrhythmia, aminotransferase (mild to moderate), hepatic hypoplasia (mild)	16.3	8.6	1-6

RIAC, Regional intra-arterial chemotherapy; SC, systemic chemotherapy.

FAM, adriamycin 40 mg/m2, mitomycin (MMC) 6 mg/m2, d1; 5-fluorouracil (5-FU), 375 mg/m2, d2-6.

GEM, gemcitabine, 1000 mg/m2, day 1, 8.

MF, MMC, 2 mg, d2, 4,6; 5-FU, 750 mg, day 1, 3, 5.

MmMC, Mitomycin C at a total dose of 18 mg/m2, day 1–5; mitoxanthrone, 6 mg/m2, day 6; cisplatin, 30 mg/m2, day 7–8.

GP, Gemcitabine 1000 mg/m2, day 1; cisplatin, 50 mg/m2, day 1. GF (GEM+5-FU):

GEM 1000 mg/m2, day 1; 5-Fu, 600 mg/m2, day 1–5.

NR, not reported

### Results of quality assessment

**Figure 2:**
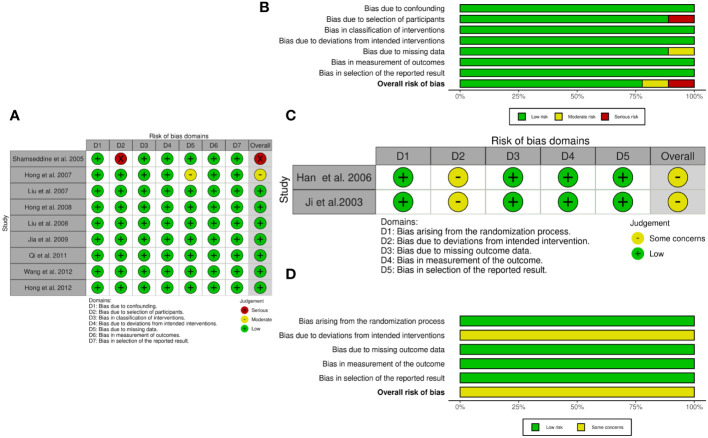
The quality assessment according to ROBINS-I and ROB 2 of each non-RCTs and RCTs. **(A)** Risk of bias ROBINS-I per study; **(B)** Risk of bias ROBINS-I per domain; **(C)** Risk of bias ROB-2 per study; **(D)** Risk of bias ROB-2 per domain.

We assessed the methodological quality of each non-RCT by using the ROBINS-I and each RCT using ROB 2. The risks of bias and corresponding ratios are summarized in **Figure 2**.

### Complete remission, partial remission, and objective response rate

**Figure 3:**
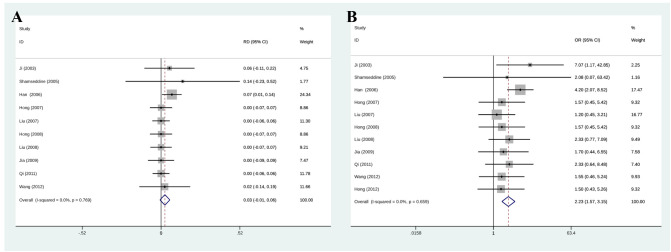
**(A)** Meta-analysis of CR. Diamonds represent pooled effects. CR, complete remission; **(B)** Meta-analysis of PR. Diamonds represent pooled effects. PR, partial remission.

**Figure 4:**
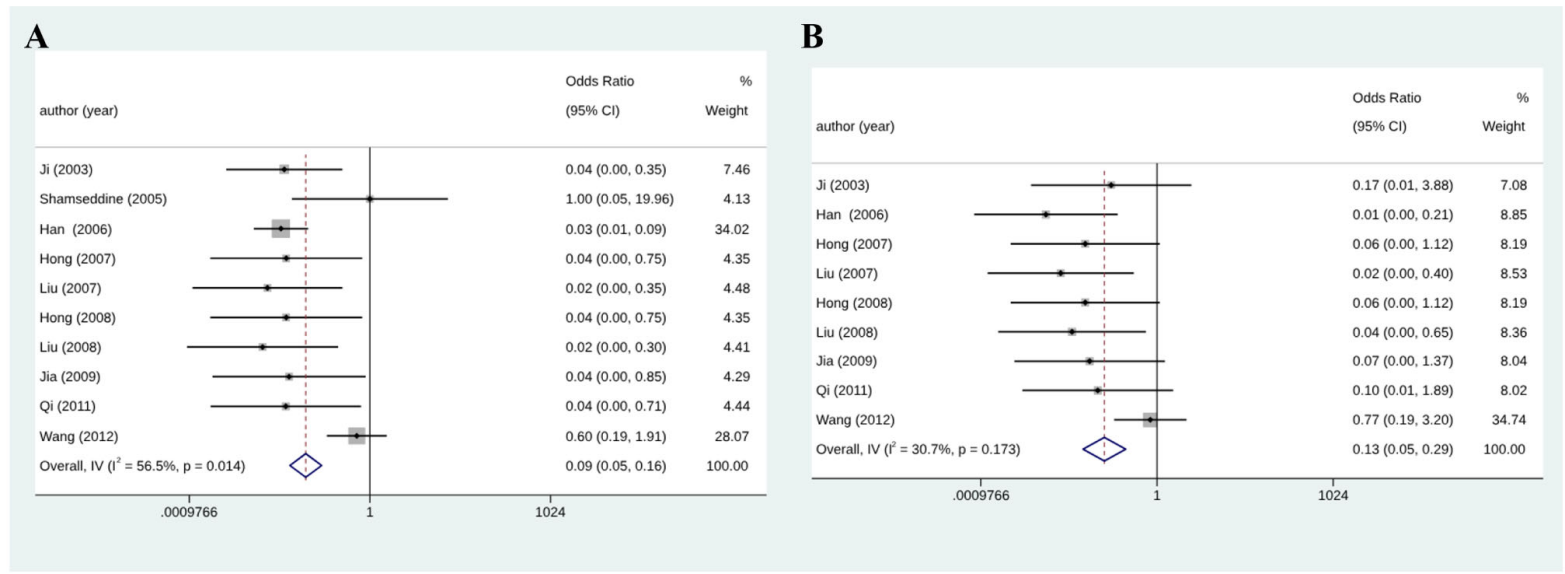
**(A)** Meta-analysis of ORR for RIAC patients; **(B)** Meta-analysis of ORR for systemic chemotherapeutics patients.

Among eleven initially selected studies (627 patients), ten were finally included in this meta-analysis of CR. **Figure 3** shows that the RIAC and systemic groups did not differ significantly for CR (RD = 0.03, 95% CI: -0.01, 0.06, I^2^ = 0%) (**Figure 3A**). However, **Figure 3B** also shows that patients treated with RIAC had better PR than those treated with systemic chemotherapy (OR = 2.23, 95% CI: 1.57, 3.15, I^2^ = 0%). In addition, according to CR and PR, the pooled ORR of RIAC patients was (OR = 0.09, 95% CI: 0.05, 0.16, I2 = 56.5%) (**Figure 4A**), while the pooled ORR of the systemic chemotherapy patients was (OR = 0.13, 95% CI: 0.05, 0.29, I2 = 30.7%) (**Figure 4B**).

### Median survival times

Ten studies (21–25, 27–29) reported that RIAC median survival times (10–21 months) were longer than for systemic chemotherapy (4.8–14 months). One study (30) reported that systemic chemotherapy median survival times (5.6 months) were longer than for RIAC (5 months). One study (26) did not report the median survival times. We tried contacting the authors but could not obtain further information. The median survival times were longer in patients receiving RIAC than those receiving systemic chemotherapy.

### Side effects

**Figure 5:**
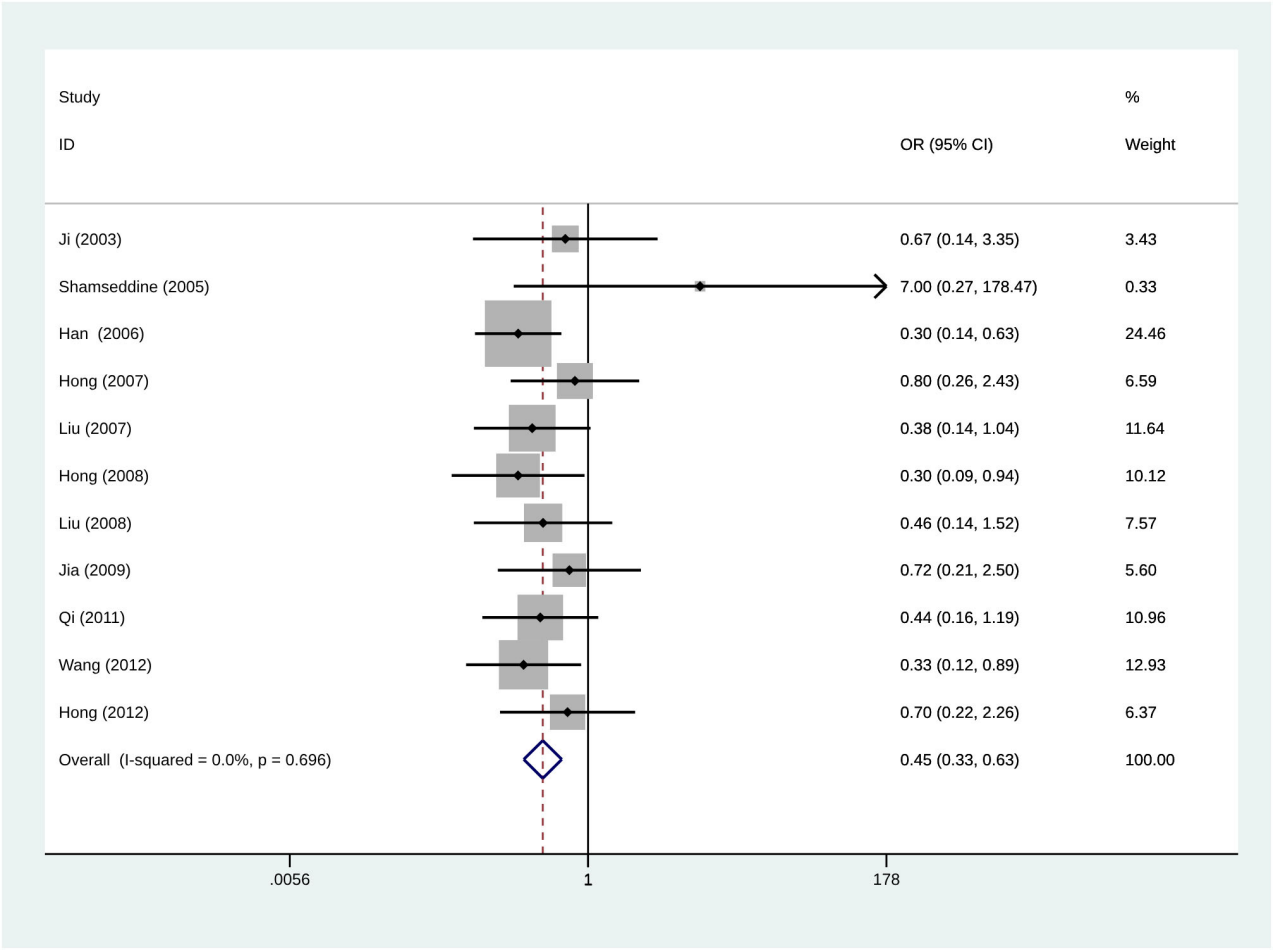
Meta-analysis of the incidence of total complications using Regional Intra-Arterial Chemotherapy or systemic administration of chemotherapeutics. Diamonds represent pooled effects.

The results in **Figure 5** show that the overall complication rate was lower in patients with RIAC than in patients receiving systemic chemotherapy (OR = 0.45, 95% CI: 0.33, 0.63, I^2^ = 0%). Common side effects included myelosuppression (leukopenia, thrombocytopenia, gastrointestinal reactions (nausea, vomiting, and diarrhea), and hepatic and renal impairment. Two studies reported severe myelosuppression in both RIAC and SC (systemic chemotherapy) groups (23, 27). One study reported severe myelosuppression and one death in the SC group (26). No deaths due to drug toxicity were reported in the RIAC group.

### Subgroup analysis

We performed subgroup analysis on each outcome by diagnostic criteria, drug, and route of administration. RIAC patients showed higher CR than the SC patients in the biopsy-proven group (RD = 0.08, 95% CI: 0.01, 0.15, I2 = 0%) and FAM group (RD = 0.07, 95% CI: 0.01, 0.14, I^2^ = 0%) (**Supplementary Figure S1**). Also, RIAC patients showed higher PR than the SC group when patients were stratified into those receiving drugs through the celiac artery (OR = 2.93, 95% CI: 1.81, 4.74, I^2^ = 4.2%), MF group (OR = 7.07, 95% CI: 1.17, 42.85, I^2^ = 0%), FAM group (OR = 2.08, 95% CI: 0.07, 63.42, I^2^ = 0%), proven group (OR = 1.81, 95% CI: 1.20, 2.71, I^2^ = 0%), and biopsy-proven group (OR = 4.07, 95% CI: 2.03, 8.14, I^2^ = 0%) (**Supplementary Figure S2**).

Also, the RIAC group showed fewer side effects than the SC group when patients were stratified in the pathologically proven group (OR = 0.48, 95% CI: 0.33, 0.70, I^2^ = 0%), biopsy-proven group (OR = 0.39, 95% CI: 0.19, 0.78, I^2^ = 71.8%), FAM group (OR = 0.30, 95% CI: 0.14, 0.63, I^2^ = 0%), GF group (OR = 0.48, 95% CI: 0.30, 0.75, I^2^ = 0%), GP group (OR = 0.44, 95% CI: 0.21, 0.96, I^2^ = 0%), drugs administered through abdominal cavity artery group (OR = 0.40, 95% CI: 0.25, 0.62, I^2^ = 0%) and drugs administered through superior mesenteric artery group (OR = 0.45, 95% CI: 0.23, 0.90, I^2^ = 0%) (**Supplementary Figure S3**).

### Sensitivity analysis

**Figure 6:**
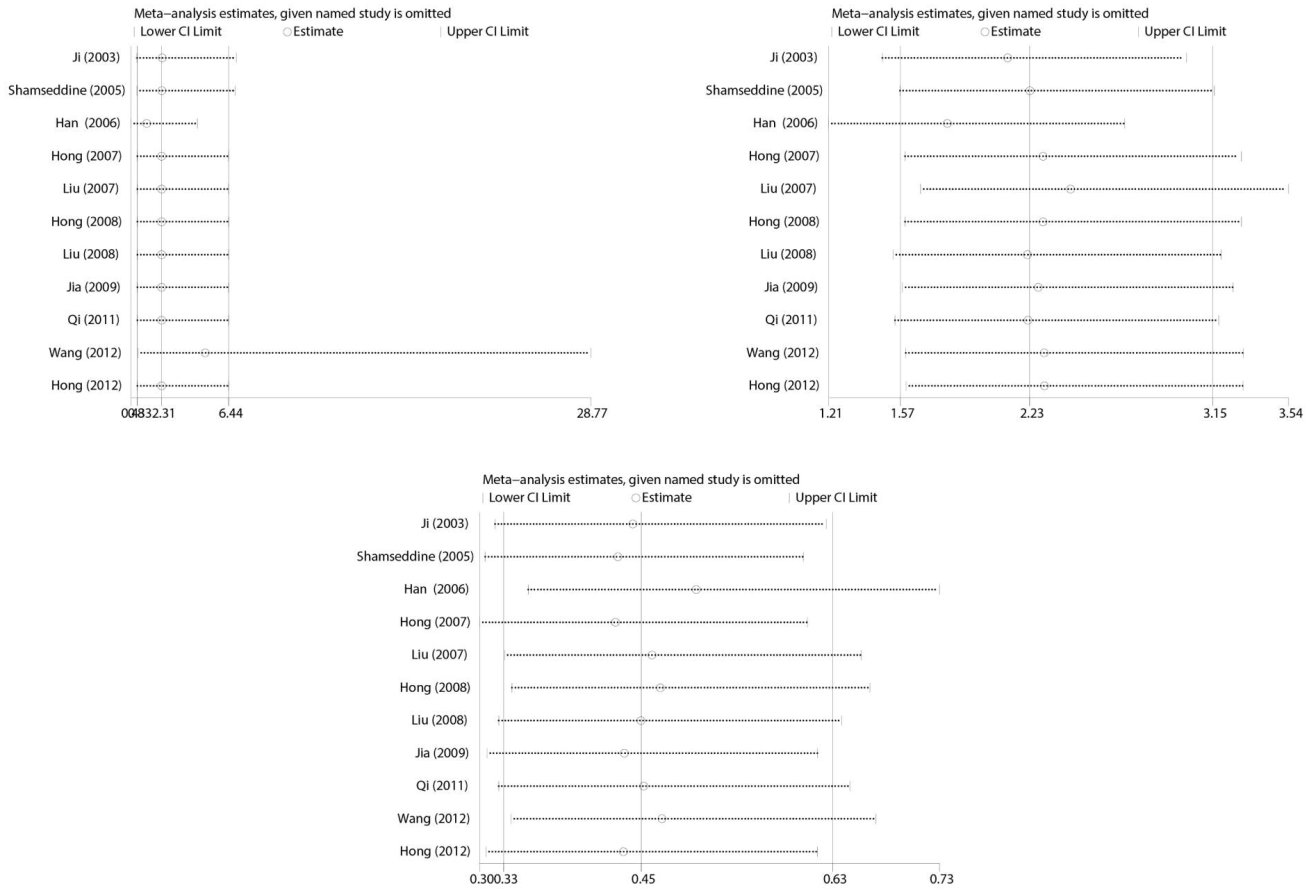
Meta-analysis of Sensitivity analysis.

Sensitivity analysis was performed to estimate the effect of each study on pooled OR by consecutive deletion of each study. The results showed no eligible study significantly influenced the pooled estimate (**Figure 6**).

### Publication bias

**Figure 7:**
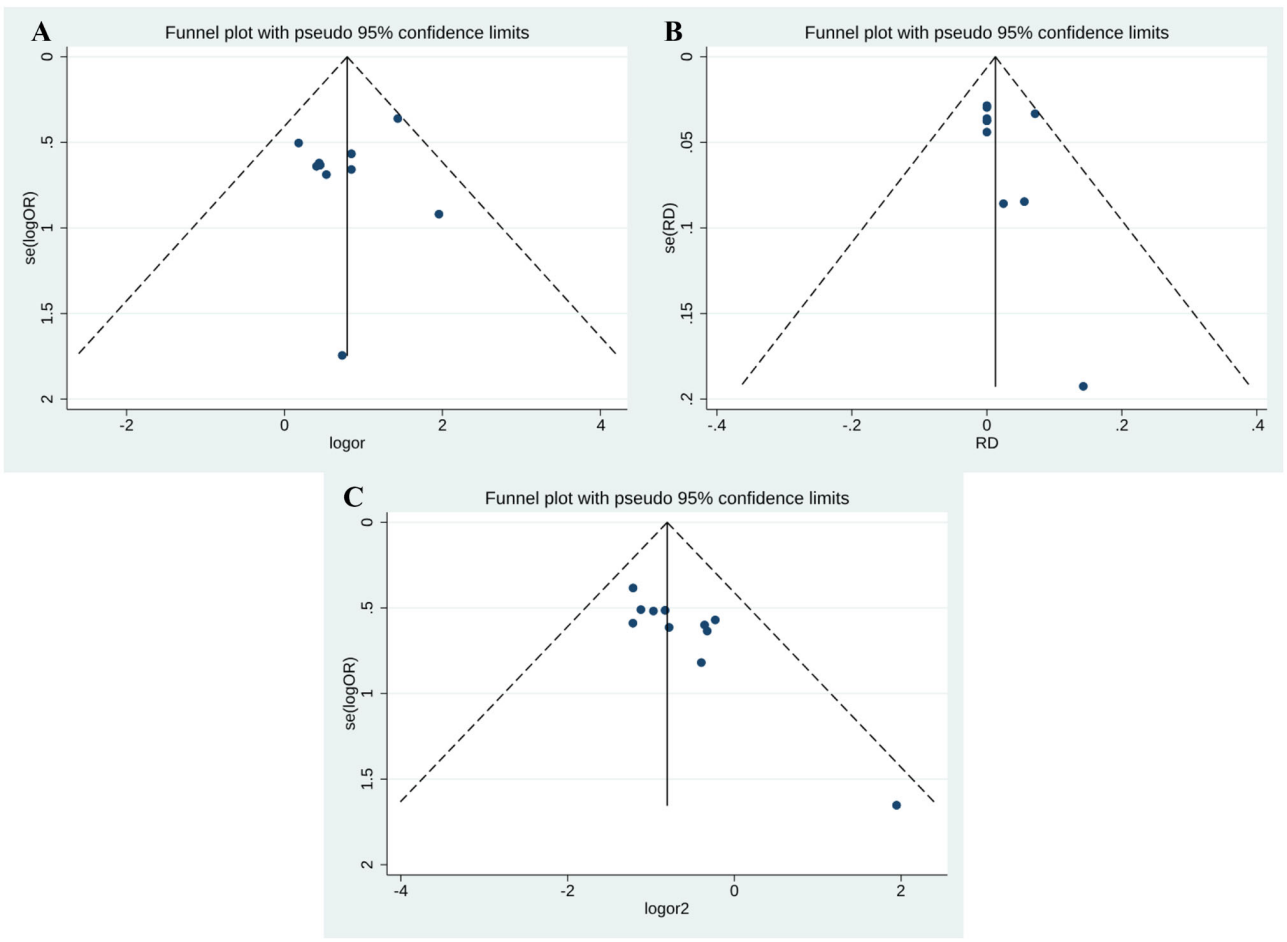
Meta-analysis of Publication bias. **(A)** CR; **(B)** PR; **(C)** ORR.

Funnel plot and Egger’s test were performed to assess publication bias among the studies. As shown in **Figure 7**, there was no evidence of publication bias for PR (Egger’s test P = 0.469) (**Figure 7A**) and CR (Egger’s test P = 0.330) (**Figure 7B**). However, side effects may be subject to publication bias (Egger’s test P = 0.002) (**Figure 7C**).

## Discussion

Our data revealed that patients who received RIAC treatment had better outcomes than those who received systemic chemotherapy, regardless of whether the treatment resulted in complete or partial remission or extended median survival time. Additionally, the incidence of side effects for patients who received RIAC was lower.

Conventional systemic chemotherapy for advanced pancreatic cancer can improve symptoms and prolong survival to a certain extent, but the overall efficacy is not ideal. Due to the drug resistance and poor sensitivity to chemotherapy, the therapeutic effect of chemotherapy on pancreatic cancer is limited (31, 32). Also, considering that pancreatic cancer has a poor blood supply and its tumor surface is often covered by a dense fibrous envelope, the effect of chemotherapeutics is limited (17). Moreover, pancreatic cancer often expresses medium to high levels of multi-drug resistance gene, which influences the chemotherapeutics effect (9, 16, 33). Therefore, increasing the concentration of tumor local drugs is necessary. Thus, changing the route of administration is often considered for these patients.

Pancreatic cancer has a dose-dependent sensitivity to local chemotherapy (34). The application of targeted arterial perfusion therapy can effectively increase the tissue drug concentration, increasing the sensitivity of tumor cells to chemotherapeutic drugs and contributing to overcoming tumor cell resistance induced by P-170 glycoprotein (9). Therefore, this method has often been applied for treating pancreatic cancer (35). Regional chemotherapy is a comprehensive treatment for pancreatic cancer. The main arterial blood supply to the pancreas comes from the trunk celiac artery and the superior mesenteric artery, so anti-cancer drugs injected through the trunk celiac artery and the superior mesenteric artery can cover the entire pancreas (17). Regional arterial perfusion chemotherapy of the pancreas can significantly increase the concentration of drugs in the pancreas, duodenum, and peripancreatic lymph nodes, enhance drug action, reduce systemic toxic and side effects, and improve the effect of chemotherapy (35, 36).

Based on the results of the present meta-analysis, we concluded that RIAC has fewer complications than systemic chemotherapy. In particular, the drug dose used for each treatment regimen was the same across studies, and RIAC had fewer severe myelosuppression events and GI reactions than systemic chemotherapy. (37) Local perfusion chemotherapy increases the blood concentration of tumor tissue, while the influence of chemotherapy drugs on other tissues, such as bone marrow tissue, liver, kidney, and gastrointestinal tract, is reduced, alleviating the toxic and side effects of systemic chemotherapy (10). Although the value of RIAC has been demonstrated, several disadvantages have limited the expansion of its clinical use. The studies included in this paper did not describe the exact length of the procedure; other studies have shown that RIAC is often more challenging to perform than systemic chemotherapy. It is also less frequently used than regular intravenous chemotherapy as the surgeon who performs it requires special training. Moreover, it is an invasive procedure that increases hospitalization time, costs, and local complications (9). However, RIAC has superior clinical benefits and fewer complications, which makes it a good strategy for advanced pancreatic cancer treatment and a good option for palliative or neoadjuvant therapy, especially in patients who do not respond to standard therapy. Generally, regional arterial chemotherapy may be more expensive than other cancer treatments, such as systemic chemotherapy or radiation therapy. However, the cost of regional arterial chemotherapy may be justified due to its potential benefits, such as higher response rates and fewer side effects compared to other treatment methods. Also, when considering the economic impact of regional intra-arterial versus systemic chemotherapy, several factors should be considered; these may include the cost of the drugs themselves, the cost of administering the treatment, the cost of any necessary hospital stays or follow-up appointments, and the potential for lost income due to time off work. Additionally, it’s important to consider the potential benefits of each treatment option in terms of overall survival, quality of life, and potential side effects. By carefully weighing these factors, healthcare providers can make informed decisions about which treatment option is most appropriate for each individual patient.

Our study has several major strengths compared with the former meta-analysis conducted in 2012 (13). First, we included 11 studies with 627 participants, while the previous study, conducted by Liu et al., was based on 5 RCTs, which included 298 participants. Also, we included four articles that were identical to Liu et al.; one article was excluded for not meeting the inclusion criteria, and 7 new articles after 2012 were included. Therefore, the result of our study may be more reliable. Second, compared with the previous meta-analysis, we used Stata software for meta-analysis; the results are more intuitive and straightforward for clinicians to understand. In the meta-analysis of the complication incidence in regional and systemic chemotherapeutics, our study yielded 0% in the heterogeneity index I^2^ (24% in the previous study); the lower I^2^ indicated a less heterogeneous population and more robust results than the former meta-analysis. Finally, the pooled CR of regional intra-arterial vs. systemic chemotherapy for treating advanced pancreatic cancer was higher than that reported by the previous meta-analysis. The different results suggested that the latest research has added new evidence to the current understanding, and RIAC is still the more effective option.

Potential limitations of this meta-analysis should also be considered. First, due to the small amount of literature in this study, the original literature is not detailed enough, and the reliability of each literature differed. Only a few studies gave a definite length of follow-up, and although the length of follow-up was consistent between the RIAC and SC groups, data on long-term prognosis are still insufficient. In addition, unpublished studies were not included in this meta-analysis, and the sample size in this study was small. Furthermore, our original literature was not randomized, and there has been an evident lack of research in recent years. Therefore, more rigorous RCTs are needed to enhance our understanding of this issue further.

## Conclusions and future directions

Based on the results of the current meta-analysis, we concluded that compared with systemic chemotherapy, RIAC has a higher PR, greater clinical benefit, and fewer complications in the treatment of advanced pancreatic cancer.

## Data availability statement

The original contributions presented in the study are included in the article/**Supplementary Material**. Further inquiries can be directed to the corresponding authors.

## Author contributions

Conception and design: YC. Administrative support: YW. Provision of study materials or patients: DY. Collection and assembly of data: YC. Data analysis and interpretation: YC. Manuscript writing: WZ. Final approval of manuscript: YC. All authors contributed to the article and approved the submitted version.

## Supplementary material

Supplementary Figure 1 Subgroup analysis of CR: **(A)** diagnostic criteria; **(B)** drug; **(C)** route of administration.

Supplementary Figure 2 Subgroup analysis of PR: **(A)** diagnostic criteria; **(B)** drug; **(C)** route of administration.

Supplementary Figure 3 Subgroup analysis of side effects: **(A)** diagnostic criteria; **(B)** drug; **(C)** route of administration.

## References

[1] Klein AP . Pancreatic cancer epidemiology: understanding the role of lifestyle and inherited risk factors. Nat Rev Gastroenterol Hepatol. (2021) 18:493–502. doi: 10.1038/s41575-021-00457-x 34002083 PMC9265847

[2] Mizrahi JD Surana R Valle JW Shroff RT . Pancreatic cancer. Lancet. (2020) 395:2008–20. doi: 10.1016/s0140-6736(20)30974-0 32593337

[3] Cao D Song Q Li J Jiang Y Wang Z Lu S . Opportunities and challenges in targeted therapy and immunotherapy for pancreatic cancer. Expert Rev Mol Med. (2021) 23:e21. doi: 10.1017/erm.2021.26 34906271

[4] Ilic M Ilic I . Epidemiology of pancreatic cancer. World J Gastroenterol. (2016) 22:9694–705. doi: 10.3748/wjg.v22.i44.9694 PMC5124974 27956793

[5] Ansari D Tingstedt B Andersson B Holmquist F Sturesson C Williamsson C . Pancreatic cancer: yesterday, today and tomorrow. Future Oncol. (2016) 12:1929–46. doi: 10.2217/fon-2016-0010 27246628

[6] Chin V Nagrial A Sjoquist K O'Connor CA Chantrill L Biankin AV . Chemotherapy and radiotherapy for advanced pancreatic cancer. Cochrane Database Syst Rev. (2018) 3:Cd011044. doi: 10.1002/14651858.CD011044.pub2 29557103 PMC6494171

[7] Zhao Z Liu W . Pancreatic cancer: A review of risk factors, diagnosis, and treatment. Technol Cancer Res Treat. (2020) 19:1533033820962117. doi: 10.1177/1533033820962117 33357065 PMC7768873

[8] Chawla A . Contemporary trials evaluating neoadjuvant therapy for resectable pancreatic cancer. J Surg Oncol. (2021) 123:1423–31. doi: 10.1002/jso.26393 33831254

[9] Laface C Laforgia M Molinari P Foti C Ambrogio F Gadaleta CD . Intra-arterial infusion chemotherapy in advanced pancreatic cancer: A comprehensive review. Cancers (Basel). (2022) 14. doi: 10.3390/cancers14020450 PMC8774130 35053614

[10] Park W Chawla A O'Reilly EM . Pancreatic cancer: A review. Jama. (2021) 326:851–62. doi: 10.1001/jama.2021.13027 PMC9363152 34547082

[11] Ishikawa T . Is it relevant that intra-arterial chemotherapy may be effective for advanced pancreatic cancer? World J Gastroenterol. (2007) 13:4306–9. doi: 10.3748/wjg.v13.i32.4306 PMC4250854 17708601

[12] Kow AWC . Hepatic metastasis from colorectal cancer. J Gastrointest Oncol. (2019) 10:1274–98. doi: 10.21037/jgo.2019.08.06 PMC6955002 31949948

[13] Liu F Tang Y Sun J Yuan Z Li S Sheng J . Regional intra-arterial vs. systemic chemotherapy for advanced pancreatic cancer: a systematic review and meta-analysis of randomized controlled trials. PloS One. (2012) 7:e40847. 22815840 10.1371/journal.pone.0040847 PMC3399885

[14] Ma N Wang Z Zhao J Long J Xu J Ren Z . Improved survival in patients with resected pancreatic carcinoma using postoperative intensity-modulated radiotherapy and regional intra-arterial infusion chemotherapy. Med Sci Monit. (2017) 23:2315–23. doi: 10.12659/msm.904393 PMC5443358 28512284

[15] Liu X Yang X Zhou G Chen Y Li C Wang X . Gemcitabine-based regional intra-arterial infusion chemotherapy in patients with advanced pancreatic adenocarcinoma. Med (Baltimore). (2016) 95:e3098. doi: 10.1097/md.0000000000003098 PMC4839930 26986149

[16] Gupta R Amanam I Chung V . Current and future therapies for advanced pancreatic cancer. J Surg Oncol. (2017) 116:25–34. doi: 10.1002/jso.24623 28591939

[17] Aigner KR Gailhofer S Selak E Aigner K . Intra-arterial infusion chemotherapy versus isolated upper abdominal perfusion for advanced pancreatic cancer: a retrospective cohort study on 454 patients. J Cancer Res Clin Oncol. (2019) 145:2855–62. doi: 10.1007/s00432-019-03019-6 PMC6800855 31506738

[18] Qiu B Zhang X Tsauo J Zhao H Gong T Li J . Transcatheter arterial infusion for pancreatic cancer: a 10-year National Cancer Center experience in 115 patients and literature review. Abdom Radiol (NY). (2019) 44:2801–8. doi: 10.1007/s00261-019-02022-2 31025067

[19] McInnes MDF Moher D Thombs BD McGrath TA Bossuyt PM Clifford T . Preferred reporting items for a systematic review and meta-analysis of diagnostic test accuracy studies: the PRISMA-DTA statement. Jama. (2018) 319:388–96. doi: 10.1001/jama.2017.19163 29362800

[20] Hong GB Zhou JX Sun HB Li CY Song LQ . Continuous transarterial infusion chemotherapy with gemcitabine and 5-Fluorouracil for advanced pancreatic carcinoma. Asian Pac J Cancer Prev. (2012) 13:2669–73. doi: 10.7314/apjcp.2012.13.6.2669 22938439

[21] Hong G Zhou J Liang B . Clinical analysis of continuous arterial infusion chemotherapy for advanced pancreatic cancer. Cancer Prev Treat Res. (2007) 34:54–6. doi: 10.3971/j.issn.1000-8578.2007.01.017

[22] Jia L Zheng J Zhang S Xie D . A comparative study of gemcitabine arterial infusion chemotherapy and peripheral intravenous chemotherapy in the treatment of advanced pancreatic cancer. Chin J Pancreatic Dis. (2009), 15–7.

[23] Liu H Li Y Huang R Huang X . Clinical observation of regional arterial perfusion of pancreas with GP scheme for advanced pancreatic cancer. Basic Clin Oncol. (2008) 21:479–81.

[24] Liu L Wang J Wang X Yan Z Liu R Chen Y . Effect analysis of arterial gemcitabine chemotherapy for advanced pancreatic cancer. Chin J Med Comput Imaging. (2007), 202–7. doi: 10.19627/j.cnki.cn31-1700/th.2007.03.016

[25] Qi X Liu D Jiang Y . A comparative study of regional chemotherapy and intravenous chemotherapy with GP protocol in the treatment of advanced pancreatic cancer. Chin J Cancer Prev. (2011) 18:1556–1558+1562. doi: 10.16073/j.cnki.cjcpt.2011.19.016

[26] Wang W Peng Z Lei X Lin Y . Comparison of the efficacy of arterial infusion chemotherapy and systemic chemotherapy in the treatment of advanced pancreatic cancer. Med J West China. (2012) 24:1730–2.

[27] Han GH Yin ZX Meng XJ He CY Zhang HB Sun AH . Prospective randomized clinical trial of two drug delivery pathway in the treatment of inoperable advanced pancreatic carcinoma. Chin J Dig Dis. (2006) 7:45–8. doi: 10.1111/j.1443-9573.2006.00243.x 16412037

[28] Ji Z Wang Y Chen X Wu T . Peripancreatic artery ligation and artery infusion chemotherapy for advanced pancreatic carcinoma. Chin Med J (Engl). (2003) 116:89–92. 12667396

[29] Shamseddine AI Khalifeh MJ Mourad FH Chehal AA Al-Kutoubi A Abbas J . Comparative pharmacokinetics and metabolic pathway of gemcitabine during intravenous and intra-arterial delivery in unresectable pancreatic cancer patients. Clin Pharmacokinet. (2005) 44:957–67. doi: 10.2165/00003088-200544090-00005 16122282

[30] Hong G Zhou J Luo J Xu L Chen Y Jiang R . A clinical study on continuous transarterial infusion chemotherapy and systemic venous chemotherapy with gemcitabine and 5-fluorouracil in treating patients with advanced pancreatic carcinoma. Chinese-German J Clin Oncol. (2007) 6:457–60. doi: 10.1007/s10330-007-0086-4

[31] Grasso C Jansen G Giovannetti E . Drug resistance in pancreatic cancer: Impact of altered energy metabolism. Crit Rev Oncol Hematol. (2017) 114:139–52. doi: 10.1016/j.critrevonc.2017.03.026 28477742

[32] Li J Wientjes MG Au JL . Pancreatic cancer: pathobiology, treatment options, and drug delivery. AAPS J. (2010) 12:223–32. doi: 10.1208/s12248-010-9181-5 PMC2844509 20198462

[33] Furuse J Shibahara J Sugiyama M . Development of chemotherapy and significance of conversion surgery after chemotherapy in unresectable pancreatic cancer. J Hepatobiliary Pancreat Sci. (2018) 25:261–8. doi: 10.1002/jhbp.547 29651809

[34] de Sousa Cavalcante L Monteiro G . Gemcitabine: metabolism and molecular mechanisms of action, sensitivity and chemoresistance in pancreatic cancer. Eur J Pharmacol. (2014) 741:8–16. doi: 10.1016/j.ejphar.2014.07.041 25084222

[35] Okusaka T Furuse J . Recent advances in chemotherapy for pancreatic cancer: evidence from Japan and recommendations in guidelines. J Gastroenterol. (2020) 55:369–82. doi: 10.1007/s00535-020-01666-y PMC7080663 31997007

[36] Liu GF Li GJ Zhao H . Efficacy and toxicity of different chemotherapy regimens in the treatment of advanced or metastatic pancreatic cancer: A network meta-analysis. J Cell Biochem. (2018) 119:511–23. doi: 10.1002/jcb.26210 28608558

[37] Jain A Bhardwaj V . Therapeutic resistance in pancreatic ductal adenocarcinoma: Current challenges and future opportunities. World J Gastroenterol. (2021) 27:6527–50. doi: 10.3748/wjg.v27.i39.6527 PMC8554400 34754151

