# Supplementary figures and images for: Regional intra-arterial vs. systemic chemotherapy for the treatment of advanced pancreatic cancer: a systematic review and meta-analysis
Source: Front Oncol. 2024 Apr 8;14:1197424. doi: 10.3389/fonc.2024.1197424 (PMC11033438; doi:10.3389/fonc.2024.1197424)

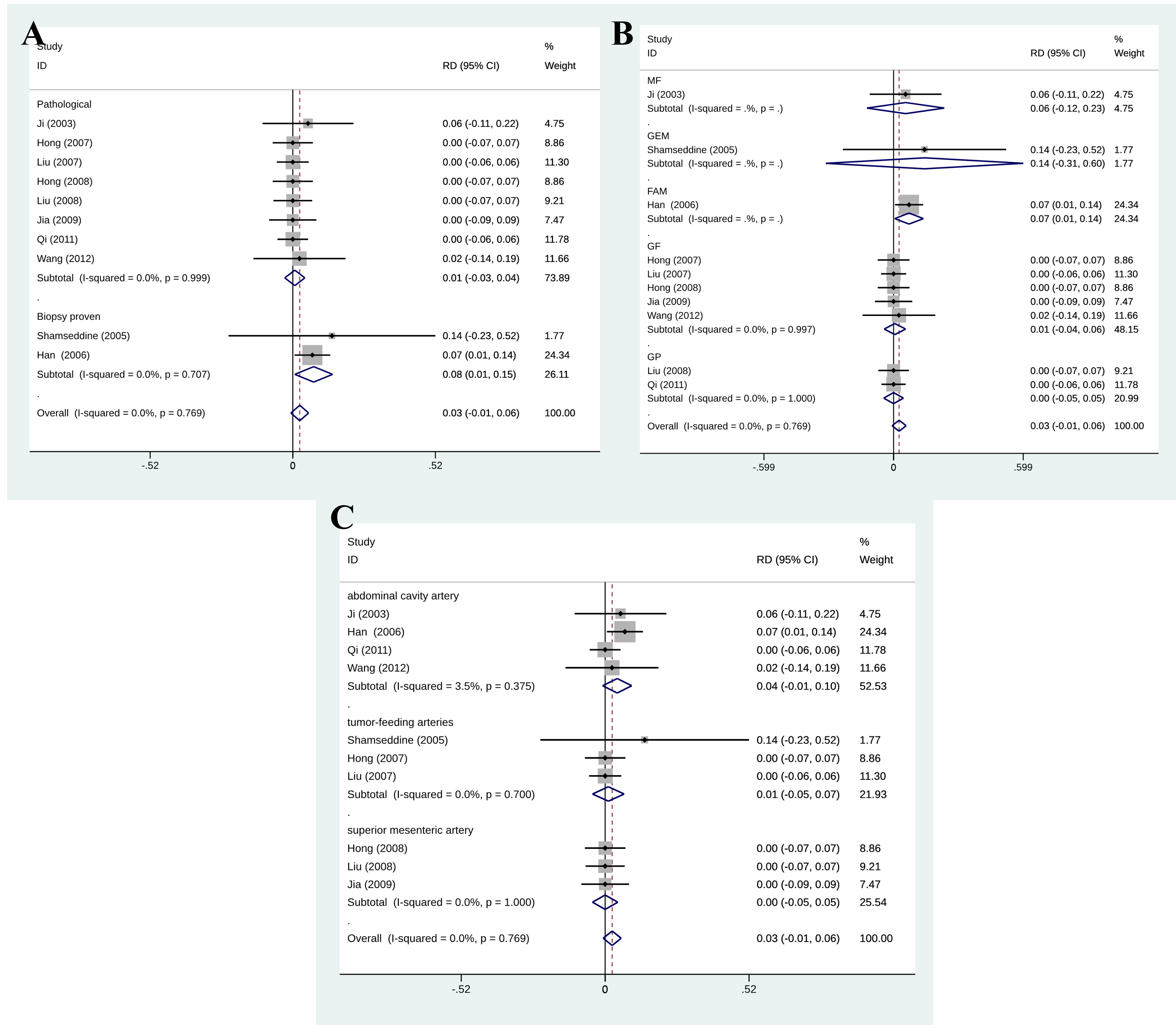

Supplement: Supplementary Figure 1 — Subgroup analysis of CR: (A) diagnostic criteria; (B) drug; (C) route of administration. [file Image_1.tiff]

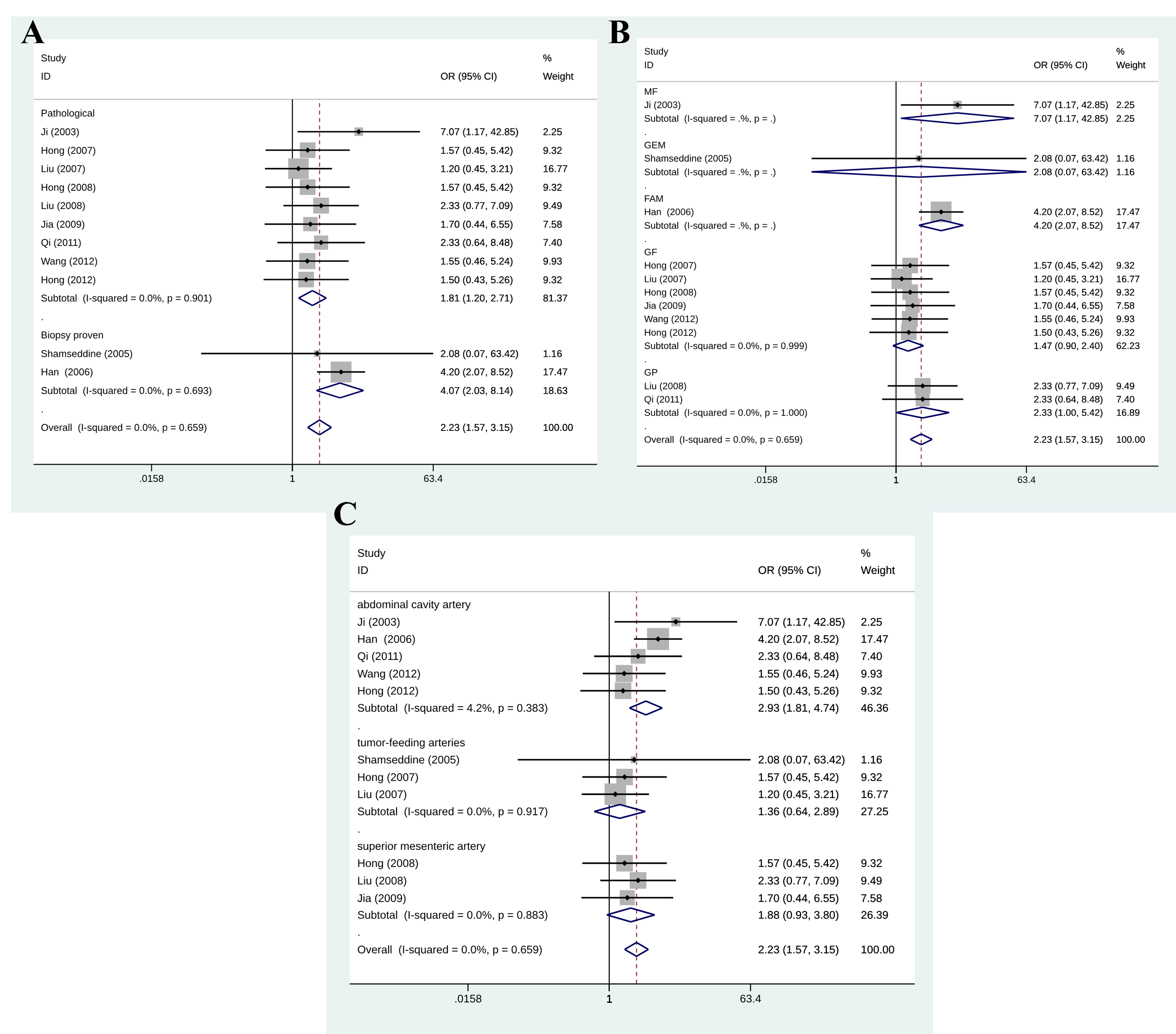

Supplement: Supplementary Figure 2 — Subgroup analysis of PR: (A) diagnostic criteria; (B) drug; (C) route of administration. [file Image_2.tiff]

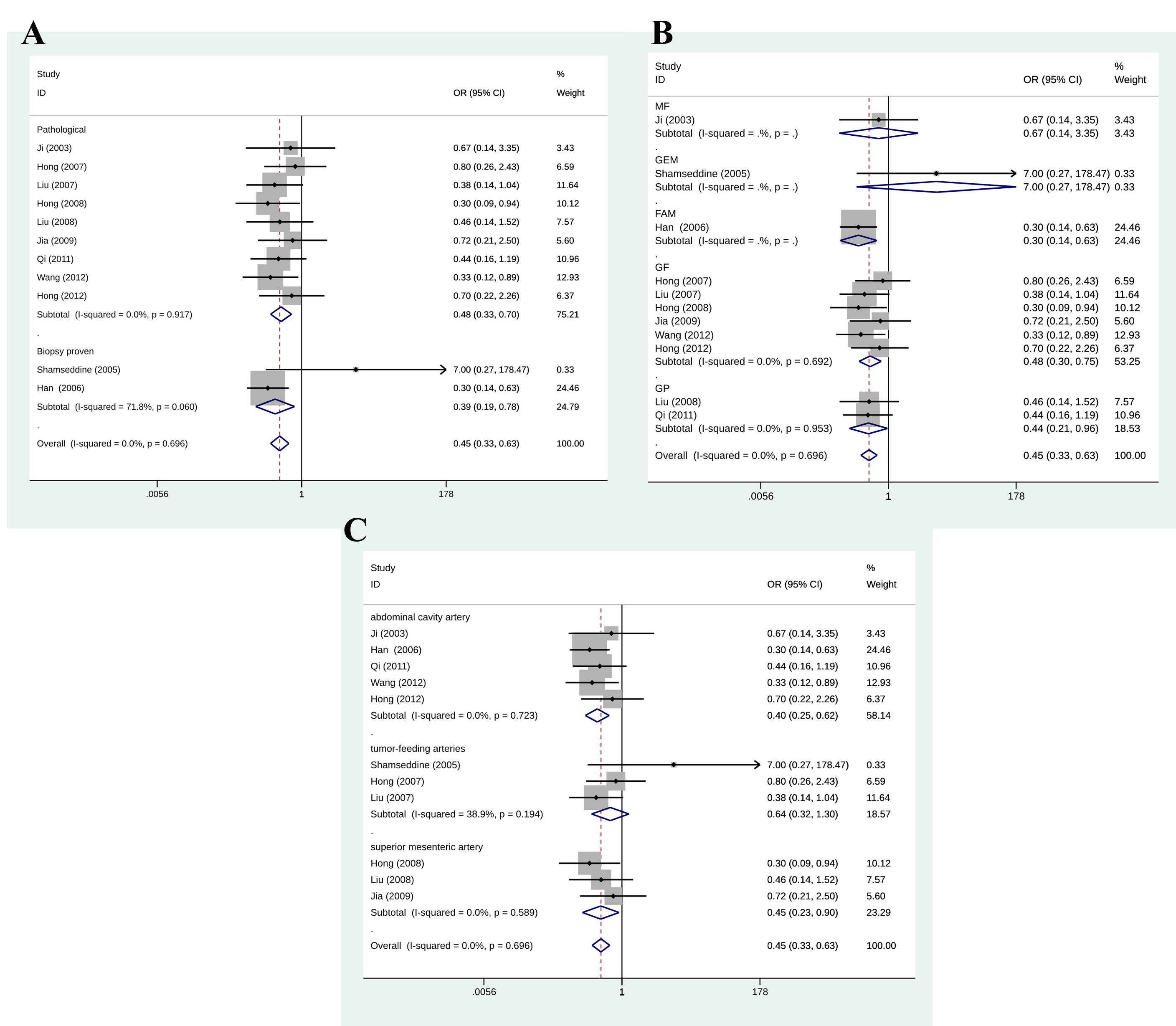

Supplement: Supplementary Figure 3 — Subgroup analysis of side effects: (A) diagnostic criteria; (B) drug; (C) route of administration. [file Image_3.tiff]
